# Sensitization, energy transfer and infra-red emission decay modulation in Yb^3+^-doped NaYF_4_ nanoparticles with visible light through a perfluoroanthraquinone chromophore

**DOI:** 10.1038/s41598-017-05350-9

**Published:** 2017-07-11

**Authors:** Haizhou Lu, Yu Peng, Huanqing Ye, Xianjin Cui, Jianxu Hu, Hang Gu, Andrei N. Khlobystov, Mark A. Green, Philip J. Blower, Peter B. Wyatt, William P. Gillin, Ignacio Hernández

**Affiliations:** 1 State Key Laboratory of ASIC and System, SIST, Fudan University, Shanghai, 200433 China; 2 Materials Research Institute and School of Physics and Astronomy, Queen Mary University of London, Mile End Road, London, E1 4NS UK; 3 Materials Research Institute and School of Biological and Chemical Sciences, Queen Mary University of London, Mile End Road, London, E1 4NS UK; 4 Division of Physics and Applied Physics, School of Physical and Mathematical Sciences, Nanyang Technological University, 21 Nanyang Link, Singapore, 637371 Singapore; 5 School of Geography, Earth and Environmental Sciences, College of Life Science, University of Birmingham, Edgbaston, Birmingham B15 2TT UK; 6 Nanoscale & Microscale Research Centre (nmRC), University of Nottingham, University Park, Nottingham, NG7 2RD UK; 7 Department of Physics, King’s College London, Strand Campus, London, WC2R 2LS UK; 8 Division of Imaging Sciences and Biomedical Engineering, King’s College London, 4th Floor Lambeth Wing, St Thomas Hospital, London, SE1 7EH UK; 9 College of Physical Science and Technology, Sichuan University, Chengdu, 610064 China; 10 Dpto. CITIMAC, Universidad de Cantabria, Facultad de Ciencias, Avda. Los Castros, s/n 39005, Santander, Spain

## Abstract

Infra-red emission (980 nm) of sub 10 nm Yb^3+^-doped NaYF_4_ nanoparticles has been sensitized through the excitation of 2-hydroxyperfluoroanthraquinone chromophore (1,2,3,4,5,6,7-heptafluro-8-hydroxyanthracene-9,10-dione) functionalizing the nanoparticle surface. The sensitization is achieved with a broad range of visible light excitation (400–600 nm). The overall near infra-red (NIR) emission intensity of Yb^3+^ ions is increased by a factor 300 as a result of the broad and strong absorption of the chromophore compared with ytterbium’s intrinsic absorption. Besides the Yb^3+^ NIR emission, the hybrid composite shows organic chromophore-based visible emission in the orange-red region of the spectrum. We observe the energy migration process from the sensitized Yb^3+^ ions at the surface to those in the core of the particle using time-resolved optical spectroscopy. This highlights that the local environments for emitting Yb^3+^ ions at the surface and center of the nanoparticle are not identical, which causes important differences in the NIR emission dynamics. Based on the understanding of these processes, we suggest a simple strategy to control and modulate the decay time of the functionalized Yb^3+^-doped nanoparticles over a relatively large range by changing physical or chemical parameters in this model system.

## Introduction

Trivalent lanthanide-doped luminescent nanoparticles have attracted considerable attention in the recent years due to their potential in optical and biological applications as a consequence of their small size and the characteristic lanthanide-based f-f transitions. These systems incorporate the lanthanide emissions’ high monochromaticity (their energy is relatively independent of the matrix), and potentially high efficiency and long lifetime together with the nanoparticles’ possibility for dispersion in aqueous, hydrophobic and polymeric environments. NaYF_4_ is one of the most studied matrices due to its low toxicity and favorable chemical and optical properties. NaYF_4_ can be produced in α (cubic) and β (hexagonal) polymorphs. With engineered lanthanide doping, it has revealed efficient up-conversion (near infra-red to visible) suitable for biological and optical applications^1–6^, the β structure showing the highest measured up-converting quantum efficiency^7^.

For both biological and other optical applications, such as lasers or telecommunications, the presence of lanthanides with NIR absorption and emission is particularly appealing. This is primarily due to the low absorbance and scattering of NIR light in silica fiber and biological tissues^8–13, 14^. Moreover, the typical long lifetime (in the order of milliseconds or hundreds of microseconds) of the NIR states of Er^3+^, Tm^3+^, Yb^3+^ and Nd^3+^ allows for 1) population inversion for lasing and amplification^13, 15^ and 2) gated detection for imaging and probing^8–12, 14^; which favors improvement of the signal to noise ratio, detection threshold and resolution (an advantage that combines with the long absorbing/emitting wavelength, away from many natural absorptions and emissions to strongly reduce backgrounds). Importantly, amplifying devices for telecommunications have also been produced based on Er^3+^-doped NaYF_4_ nanoparticles in polymer matrices^13, 15^. Among the NIR emitting lanthanides, Yb^3+^ is interesting due to its emission at an energy above the silicon bandgap and its relatively high oscillator strength and coupling to Er^3+^, which favors its use in pumped Er^3+^-based systems. In addition, Yb^3+^-based materials are used for high power lasers for sensing and industrial applications^16^.

It is known that due to Laporte’s rule, the oscillator strength of the f-f transitions of the lanthanide is limited, and thus, overall absorption (and consequently photoluminescence (PL) intensity) is decreased in comparison to optical transitions in other systems such as fluorescent molecules or semiconductors. Amongst potential solutions to increase the lanthanide excitation and emission, co-doping of the host nanoparticle matrix with other ions has been employed^4, 13^. When the aim is to increase the absorption cross-section by means of indirect excitation in another center and subsequent energy transfer to the emitting lanthanide, this is termed sensitization. In addition, surface passivation using core/shell structures have been verified as an effective way to minimize the surface quenching of lanthanide doped nanoparticles^17^. Recently, plasmonic enhancement with noble metal such as Ag and Au have been proposed and verified as a means of enhancing the local excitation field and emission rate^14, 18^.

An interesting and often successful sensitization strategy includes the incorporation of organic chromophores in ligands chelating the NIR-emitter which can transfer the absorbed energy to the lanthanide’s states (‘antenna effect’). However, exposure of the NIR-emitting lanthanides to an environment rich in C-H, O-H or N-H groups^19, 20^ (as is usual in organic compounds) results in strong vibrational quenching of the emission, due to the comparable energies of the gaps and the 3rd-5th overtones of hydrogenated groups’ vibrational quanta^19, 20^. A number of alternatives, including fluorination^21, 22^ or chlorination^12^ of the organic groups have been proposed, some of them resulting in considerable increases of the emission with relatively high emission efficiencies and correlated long lifetimes﻿﻿ allowing, for instance, NIR to visible ﻿upconversion in organic environments^22^.

The possibility of attaining organic chromophore-mediated sensitization of NIR-emitting lanthanides in inorganic matrixes, including nanoparticles, has been recently demonstrated^6^, including Yb^3+^-based NIR-to-visible up-conversion^23^. Visible lanthanide emitters^24^ can also be sensitized, similarly, upon higher energy excitation within the ultra-violet range, which can be regarded as a reciprocal process to sensitization of conjugated organic dyes from quantum dots^25^. Functionalization of nanoparticle surfaces with organic chromophores allows coupling the enhanced absorption of the chromophores, similar to the organic complexes described above, but retaining the relatively long lifetime/low quenching rates for NIR-states, as well as the nanoparticles’ dispersion and processability properties. The lanthanide ions inside the inorganic nanoparticles are relatively isolated from the surroundings, and thus less quenched in aqueous or other hydrogenated solvents, which may favor a longer lifetime for ‘wet’ applications^24^.

Importantly, the use of red-shifted chromophores in sensitized lanthanide systems is desirable over UV sensitization^9, 11, 12, 26, 27^. This is not only due to the advantages of employing low-cost excitation sources but also due to the more favorable coupling between the organic and lanthanide levels, as well as increased penetration in tissues and reduction of backgrounds or degradation of polymer hosts or surrounding environment.

Here we show that monodisperse, sub 10 nm Yb^3+^-doped α-NaYF_4_ nanoparticles can be sensitized by the corresponding deprotonated 2-hydroxy-perfluoroanthraquinone (1,2,3,4,5,6,7-heptafluro-8-hydroxyanthracene-9,10-dione) chromophore with visible excitation wavelength in the 400–600 nm, and show an extraordinary increase of the NIR emission intensity. This compound has been employed for sensitization of NIR-emitting lanthanides in tetrakis complexes in which it acts as a chelating ligand^28^ and in particular in the telecommunications range^13, 15, 29^. The complexes of this fluorinated ligand showed a relatively long lifetime due to the lack of hydrogen (but still only in the range of tens of microseconds) and moderate enhancement of the luminescence properties. In this work we employ this combination of chromophore, host and lanthanide to investigate the energy transfer pathways in the hybrid inorganic/organic composite material, and to explore the possibility of increasing the lifetime of the perfluorinated ligand-capped nanoparticle with respect to the pristine one. We have performed a spectroscopy study including dynamic measurements as a function of the excitation wavelength and pulse length, paying particular attention to the quenching mechanisms. We observe a strong dependence on chromophore to nanoparticle ratio as well as the excitation power/pulse which relates to the surface-to-core of the nanoparticle energy migration and can be employed to modulate the Yb^3+^ average lifetime. The demonstration of this energy migration, lifetime manipulation and energy transfer is an important contribution towards understanding and using these systems in applications^30^. In particular, the excitation dynamics and the role of diverse core-shell structures are the subjects of intense research in the fields of luminescent nanoparticles^31, 32^ and our work provides insight into the external parameters needed to understand it and control it.

## Results and Discussion

**Figure 1:**
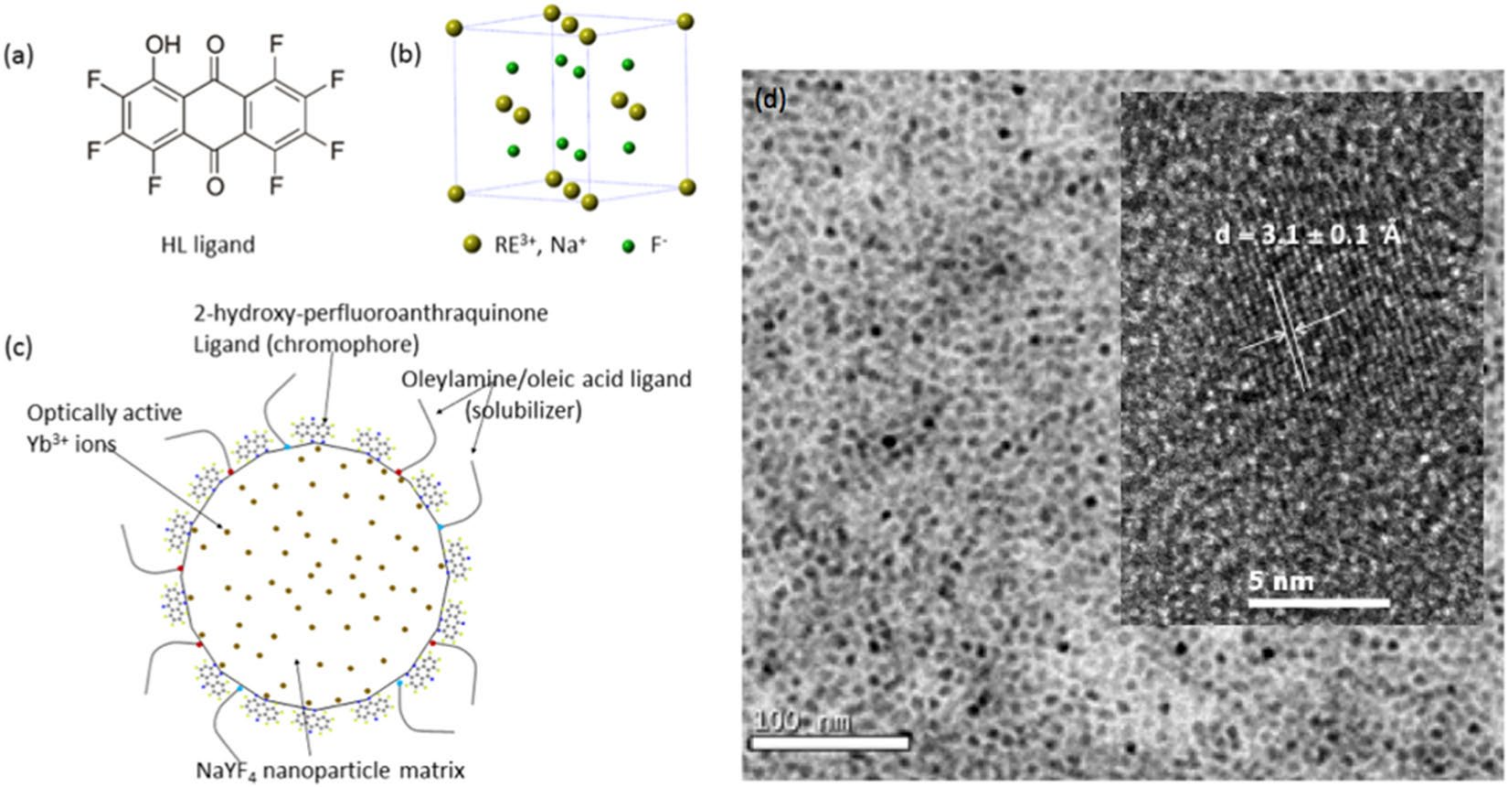
(**a**) Scheme of 2-hydroxy-perfluoroanthraquinone neutral ligand; (**b**). Cubic NaYF_4_ structure; (**c**). Chromophore-capped Yb^3+^-doped NaYF_4_ nanoparticles (proportions of the components’ sizes are approximately respected; (**d**). TEM and HRTEM (inserted) images of the 10% Yb^3+^-doped NaYF_4_ nanoparticles. The observed interplanar distance is given and found in agreement with that expected for the (1 1 1) plane.

The synthesis of Yb^3+^-doped NaYF_4_ nanoparticles and the functionalization of ligand capped Yb^3+^-doped NaYF_4_ are described in detail in the Methods section. Figure 1a shows the chemical structure of the 2-hydroxy-perfluoroanthraquinone chromophore, which was synthesized according to refs 28 and 33. The measured XRD pattern (supplementary information (SI)) of the solid residue matches the corresponding alpha (cubic, Z = 2, a = 5.47 Å) phase of NaYF_4_ (Fig. 1b), and shows the corresponding broadening of the peaks due to the small particle size. Figure 1c is a schematic picture of the ligand capped nanoparticles. Transmission electron microscopy images (Fig. 1d) reveal that the sample consists of monodisperse near-spherical monodomain nanoparticles of the Yb^3+^-doped NaYF_4_ with an average size of 6.3 nm ± 1.9 nm. The particles are slightly smaller (5.2 nm ± 2.0 nm) for 20% Yb^3+^ content than for 10% Yb^3+^.

**Figure 2:**
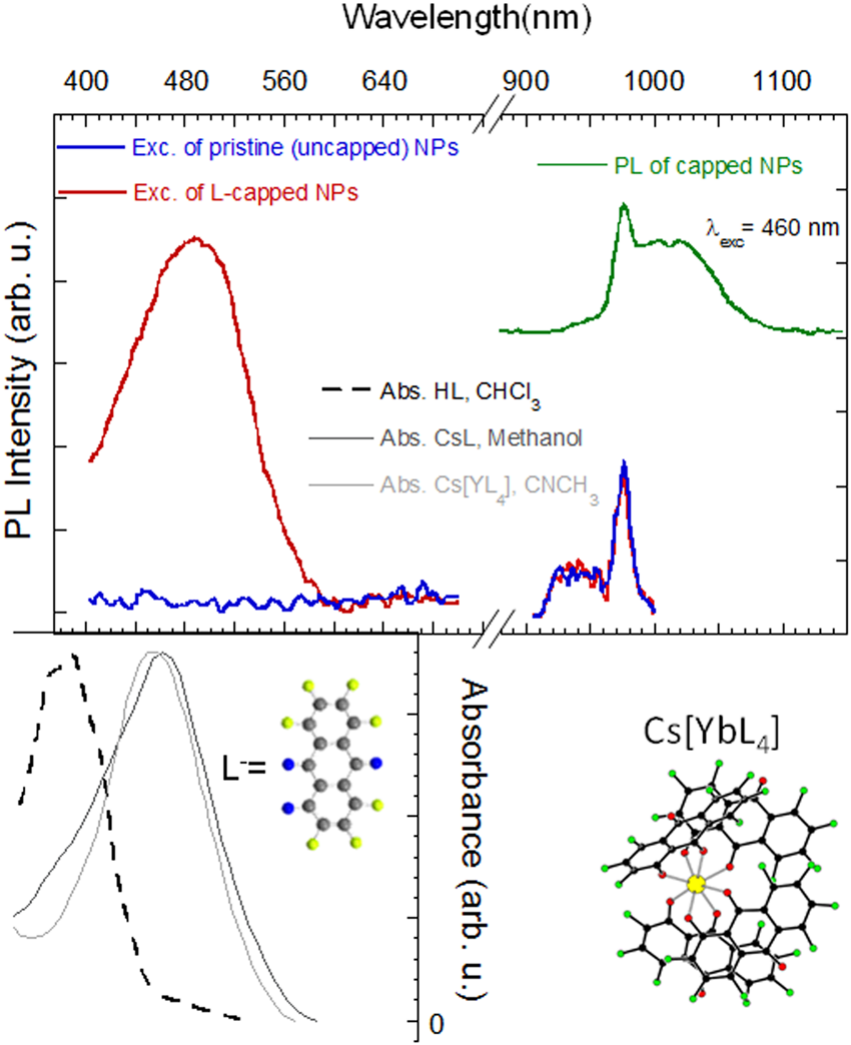
Green curve: NIR emission spectrum of 2-hydroxy-perfluoroanthraquinone-capped 10% Yb^3+^-doped NaYF_4_ nanoparticle at 460 nm excitation. Blue, red curves, resp.: excitation spectra of pristine (only Oleic-Ac/Oleyl-Am-solubilized) and functionalized (chromophore/nanoparticle suspension concentration ratio of 0.44) 10% Yb^3+^-doped NaYF_4_ nanoparticles for the 1030 nm emission. The spectra are normalized to the IR band. Dotted curve: absorption spectrum of the 2-hydroxy-perfluoroanthraquinone (LH) solution (chloroform). Gray curves: absorption of the CsL solution (methanol) and Cs[YbL_4_] complex (acetonitrile). All spectra are given on the same wavelength scale. The structure of the ligand and complex is schematically represented.

Figure 2 shows the NIR PL spectrum of the chloroform suspension of 2-hydroxy-perfluoroanthraquinone functionalized 10% Yb^3+^-doped NaYF_4_ nanoparticles at 460 nm OPO excitation. It shows the characteristic emission of Yb^3+^ arising from the ^2^F_5/2_ → ^2^F_7/2_ transition peaking at ~976 nm (~1.26 eV). A less intense, but similar photoluminescence is observed for the 20%-doped sample at similar conditions. Figure 2 also shows the excitation spectra at λ_em_ = 1030 nm for the pristine (only Oleic-Ac/Oleyl-Am-solubilized) 10% Yb^3+^-doped NaYF_4_ nanoparticles (0.35 mg/ml total concentration of the suspension) and those capped with the anthraquinone derivative chromophore (as prepared for the same nanoparticle concentration of the suspension, and a chromophore/nanoparticle suspension concentration ratio of approximately 0.44). Besides the corresponding Yb^3+^-based excitation in the NIR range corresponding to the reciprocal transition ^2^F_7/2_ → ^2^F_5/2_ in the 970 nm range, the functionalized nanoparticles show an additional broad band in the 400–600 nm range. Importantly, this band does not correspond to that of 2-hydroxyperfluoroanthraquinone in chloroform, but is comparable to the absorption band of the deprotonated 2-hydroxy-perfluoroanthraquinone (Fig. 2) and also related to the excitation band of the corresponding Er^3+^ or Yb^3+^ complexes of the same ligands^28, 33^, (SI). This suggests corresponding bidentate binding of the ligand at the cation site and strongly implies that the chromophore functionalizing the nanoparticle is capable of acting as a sensitizer for the Yb^3+^, which would otherwise show no excitation in the visible range as is the case in the excitation spectra of the pristine nanoparticles.

**Figure 3:**
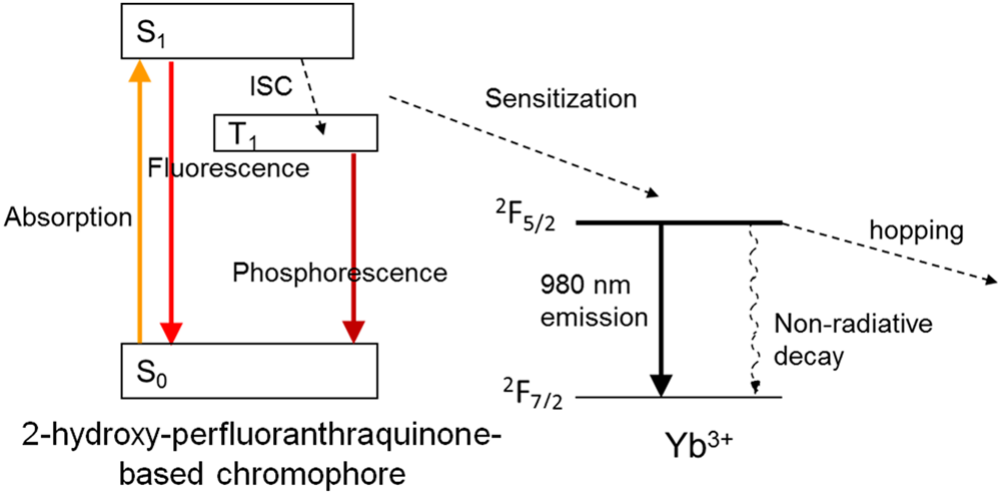
Simplified schematic representation of organic-chromophore mediated Yb^3+^ sensitization process (Jablonski diagram): S_0_: ground state of chromophore, S_1_: excited singlet state, T_1_: excited triplet state, ISC: inter-system crossing. Energy (vertical) scale is not quantitative.

The energy transfer process between organics-based states and those of the Yb^3+^ (Fig. 3) typically involves absorption in the chromophore’s singlet state (allowed optical transition) plus direct singlet to lanthanide energy transfer or triplet-based energy transfer involving a previous intersystem crossing step. Intersystem crossing causes the creation of a long-lived triplet (typically hundreds of microseconds or milliseconds, in the absence of other quenching interactions) in the organic chromophore at the expense of the short-lived singlets (typically nanoseconds or tens of nanoseconds) via some spin mixing process. The sensitization of the Yb^3+^ ions from the organics may involve energy transfer via Dexter or Förster interactions^34, 35, 36^. In the case of soft donor ligands, it may also occur via organic-Yb^3+^ charge transfer^37^, which requires corresponding intermediate metal-ligand steps involving the Yb^2+^ transitional state. For low excitation energies, as in the present case, the first mechanism (direct chromophore-Yb^3+^) is expected, and the magnitude of the transfer is increased with the overlap of the energies of the relaxed chromophore states with those of the Yb^3+^ excitation, at 1.2 eV. In this case, the relatively longer-lived triplets created by the intersystem crossing, favored by the heavy atom effect as a consequence of fluorination, may contribute positively to the coupling and subsequent energy transfer. Moreover, the relatively low energies (long wavelengths) of the ligand-based triplets emission as obtained from the Cs^+^ complex of the 2-hydroxyperfluoroanthraquinone ligand (see SI, Figure S3) suggest that triplets may participate in the sensitization energy transfer process. Nevertheless, discrimination between the exact sensitization mechanism (exchange or multipolar-based, singlet or triplet-based) is out of the scope of this paper, and usually done on the basis of transfer time and distance dependence; in the case of surface functionalized nanoparticles in solution this would become extremely difficult.

Importantly, the presence of ligand-based PL at 405 nm excitation (see SI, Figure S3), demonstrates that a portion of the singlets is still emitting and thus not all the primary excitation reaches the Yb^3+^ ions. Although this implies that the sensitization process is limited, this is not necessarily undesirable, depending on the application, as the broad band extending from 520 to 750 nm may allow, for instance for dual visible/NIR optical uses.

The quotient of the areas of the ligand- and Yb^3+^-based excitation spectrum bands represents an estimation of the sensitization efficiency. In the case represented in Fig. 2, Yb^3+^-based NIR luminescence is 7.5 times more intense upon chromophore excitation, than in direct excitation. We observe a dependence of this number with the total preparation concentration resulting in an overall decrease at high concentrations (SI, Figure S4).

**Figure 4:**
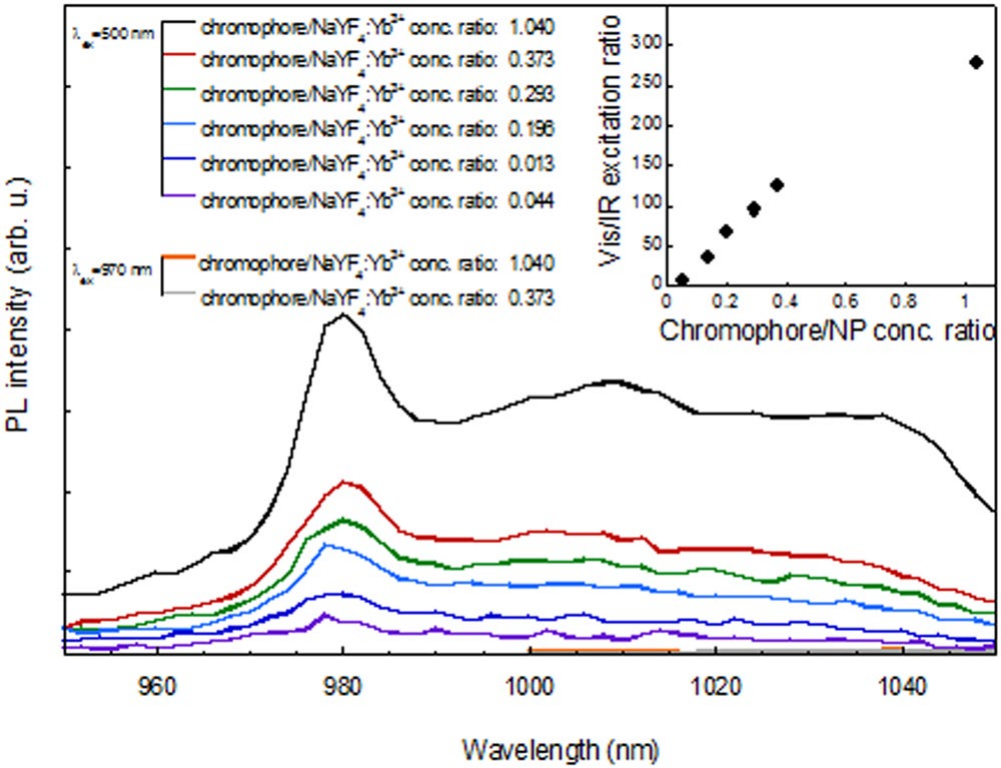
NIR emission spectra of 2-hydroxy-perfluoroanthraquinone-functionalized Yb^3+^-doped (10%) NaYF_4_ nanoparticle prepared with different chromophore to nanoparticle suspension concentration ratios (by varying the amount of chromophore for a constant nanoparticle suspension concentration of 0.182 mg/ml). The inset shows the corresponding excitation ratios, derived from the total PL intensity at 1030 nm, for visible (400–700 nm) excitation divided by the intensity for IR excitation (890–1020 nm), see Fig. 2.

Apart from the total concentration of the functionalized nanoparticles, the measured sensitization derived from the PL intensity under direct and indirect excitation depends on the chromophore to nanoparticle suspension concentration ratio. Figure 4 shows the NIR PL spectrum for various concentration ratios, for a constant concentration of the nanoparticle suspension (0.182 mg/ml), under indirect excitation. Since we observe a minor increase of the PL upon direct excitation (which we assign to the increase in the protection of the nanoparticles from the hydrogenated environment, upon denser coating), but it is considerably smaller than upon excitation in the chromophore, we interpret this result as a significant increase in the sensitization efficiency. Higher ligand to nanoparticle ratios allow for an enhanced sensitization at the given excitation conditions (Fig. 4, inset). The corresponding measured quotient of visible/IR excitation areas for the most intense infra-red intensities reaches a value around 270 (see Fig. 4, inset), for the preparation with chromophores/nanoparticle suspension concentration ratios of approximately 1.04. On the basis of the corresponding average particle size, structure and composition, this represents a coating of around 900 chromophore ligands per nanoparticle and approximately 5.5 chromophore per Yb ion. This is a dense, but not necessarily total coating, as the number of cations at the surface is ~1300. We must note here the additional presence of Oleic-Ac and Oleyl-Am ligands at the particle surface, and the fact that we are assuming perfect spherical shape despite the moderate deformation observed.

Although the sensitization of IR-emitting lanthanides through organic chromophores in the form of metal complexes and metal-organic frameworks and polymers has been the subject of many studies, few hybrid organic-inorganic systems showing this phenomenon have been described. Hybrid composite systems benefit from the relatively large IR emission yields of the lanthanide in the inorganic matrix while the multiphonon deactivation channels caused by the organic environment are kept away. In particular, nanoparticles of cubic NaYF_4_ doped with Yb^3+^ or doped with Nd^3+^ and functionalized with tropolone (2-hydroxy-2,4,6-cycloheptatrien-1-one) showed lanthanide-based NIR emission upon UV-blue excitation of the organic proving this strategy was possible by tailoring at the nanoscale. Quantifications of the enhancement of the optical properties through functionalization with organics in Yb^3+^ systems are scarce. To the best of our knowledge, the only reported figure can be estimated at an approximately 60 times increase of the excitation of Yb^3+^ through sensitization of β-NaYF_4_ doped with 20% Yb^3+^, 2% Er^3+^, functionalized with the organic dye IR-810 (we have derived this number from the estimated enhancement of the excited Yb^3+^ population from the published data^23^, in which the authors report enhanced UC in the hybrid system upon excitation in the dye). Thus, our system produces the highest increase to date and represents the use of a novel strategy, in which a red shifted perfluorinated coating is employed. Moreover, our system provides an interesting insight into the NIR PL dynamics.

**Figure 5:**
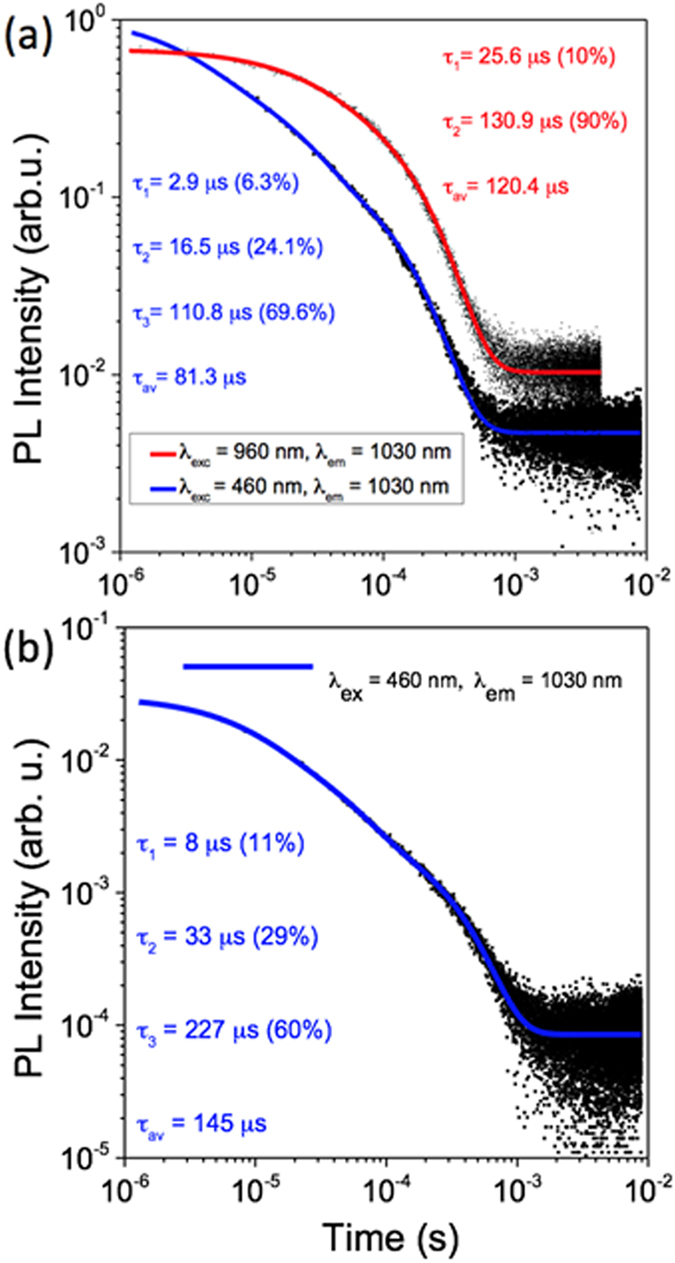
(**a**) Decay curves of 2-hydroxyperfluoroanthraquinone-capped 10% Yb^3+^-doped NaYF_4_ nanoparticles (chromophore/nanoparticle suspension concentration ratio of 0.44) with 460 nm and 960 nm OPO excitations. (**b**) I(t) decay curve and fittings for the 1030 nm emission at the 460 nm OPO excitation for the 0.37 mg/ml chromophore functionalized nanoparticle at the high chromophore/NaYF_4_: Yb^3+^ nanoparticles suspension concentration ratio of 1.04. The numbers in the brackets are the percentage contribution to the average lifetime.

Figure 5a depicts the I(t) decay curves for the 1030 nm emissions, at 460 nm and 960 nm excitations using 7 ns pulses from an OPO, for the functionalized 10% Yb^3+^-doped NaYF_4_ dissolved in chloroform solution (0.35 mg/ml, chromophore/nanoparticle molar ratio of approximately 0.025). No differences are observed along the given emission spectra but the intensity decay curves are considerably different for the different excitation conditions. The decay of the NIR-excited Yb^3+^ emission is fitted with a double exponential curve, yielding an average lifetime of 120 μs. However, the Yb^3+^ emission decay curve obtained at visible excitation shows a considerably shorter average lifetime (81 μs) and tri-exponential behavior, with two of the components being ~3 and ~17 μs, considerably shorter than any of those obtained for the NIR excitation-based emission. Interestingly, lifetimes obtained upon both excitation conditions are considerably longer than those obtained in the case of 20% Yb^3+^ content. The decrease in lifetime correlates with the differences in emission intensity and is due to significant concentration quenching^7, 14, 22, 38^ for high Yb^3+^ concentrations.

It is understood that a system described by a multiexponential decay involves different emitting centers, each showing different environments in terms of geometry or neighboring transferring sites, which results in different population and/or emission routes^17, 35, 38, 39^. In systems showing high surface to volume ratio (and particularly in those in suspension or embedded in another medium), it is usually inferred that short lifetimes correspond to emitting centers near the surface, and thus showing a more distorted geometry, or more exposed to quenching effects of the environment, while more protected, inner emitters show longer lifetimes^30^.

In view of the above, we assign the short lifetimes of the chromophore-excited emission to emitting Yb^3+^ ions near the surface. This includes the very short component of 3 μs (representing 6% of the total emission) and the 17 μs emission (24%). They correspond to given populations of Yb^3+^ ions which 1) are excited via energy transfer from the chromophore and 2) are exposed to hydrogenated molecules such as the chloroform solvent, Oleic-Ac, Oleyl-Am, other organic residues, or even the chromophore itself, which may cause a decreased lifetime of the Yb^3+^ ions, as in the case of the tetrakis complexes^33^.

Evidently, the same number of surface-based Yb^3+^ ions exists in the functionalized nanoparticle with 960 nm OPO excitations. In principle similar lifetimes could be expected irrespective of excitation conditions. However, we must observe the fact that a different population of Yb^3+^ ions is probed when the 10% Yb^3+^-doped NaYF_4_ nanoparticles are excited in the corresponding Yb^3+^ NIR absorption bands (960 nm) with respect to the ligand-based excitation (460 nm). At NIR excitation, this primarily excited population includes a considerably smaller ratio of Yb^3+^ ions at the surface than in the inside of the nanoparticle, as for the given average particle the proportion of Yb^3+^ atoms within a lattice near the surface of the particle to those more than a cell inside the volume of the particles is significant, but not major. When excited at the ligand absorption, the primarily excited population of Yb^3+^ ions are at the surface (nearly all or all at the surface). Consequently, shorter lifetimes dominate for ligand-based excitations while longer lifetimes do so under direct Yb^3+^ excitation (see Fig. 5). Non-negligible excitation hopping and diffusion among the Yb^3+^ with different lifetimes correspondingly distributed within the nanoparticle is also an important factor modulating the excitation dynamics, as we explain below.

These considerations are confirmed in view of the measured lifetime of the pristine (only Oleyl-Am/Oleic-Ac capped) 10% Yb^3+^-doped NaYF_4_ with 960 nm OPO excitation. At this excitation (direct excitation into the Yb^3+ 2^F_5/2_ state), the decay time of pristine and chromophore capped nanoparticles is the same, as they are exposed to similar organic and hydrogenated impurities (except, obviously, the anthraquinone derivative ligand, but this low chromophore/nanoparticle suspension concentration ratio represents around 35 units per particle, resulting in a poor protection from the environment). It is also important to remark that the ^2^F_5/2_ → ^2^F_7/2_ Yb^3+^ emission at 10% doping is strongly affected by energy transfer processes between the Yb^3+^ ions themselves (hopping). Consequently, the initially excited population may indeed be different from the emitting population (apart from a considerable reduction of the emission lifetime with respect to the purely radiative figure due to the excitations diffusing to quenching sites, as observed in the case of 20% Yb^3+^ doping). The above results suggest that in the ligand-based excitation experiments, excitation diffuses from the surface towards the inside of the nanoparticles and quickly reaches relatively protected Yb^3+^ ions, inside the particle. The opposite occurs for Yb^3+^ direct excitation experiment, when the excited population changes (statistically) from an initially more protected population diffusing its excitation to more exposed Yb^3+^ with a shorter lifetime.

The decay curve of the ligand-capped Yb^3+^-doped NaYF_4_ nanoparticles in this chromophore/nanoparticle suspension concentration ratio regime at chromophore-based excitation is significantly dependent on the shape of the excitation pulse. While the OPO pulse excitation at 460 nm (with an average energy of ~600 μJ/pulse, 7 ns pulse width) causes a tri-exponential decay, the decay curve with a 405 nm diode excitation (80 mW) with pulse widths higher than 1 μs can be described with two lifetimes, the components and its relative contribution changing with the pulse widths (see Table 1).

**Table 1:** Lifetime summary with different excitation pulse length (modulated 405 nm excitation, 80 mW peak power) for a chromophore/NaYF_4_: Yb^3+^ nanoparticles suspension concentration ratio of 0.44.

Pulse length [μs]	1	100	1000
τ_1_ (μs)	10.7	15.3	17.1
τ_1_%	26%	16%	11%
τ_2_ (μs)	64.5	113.6	137
τ_2_%	74%	84%	89%
τ_av_ (μs)	50.5	98.2%	124.0

Table 1 shows that longer excitation pulses in the organic ligand cause an increase of the individual lifetime components (which are considerably more dominant in the longer component) and an increase of the contribution of longer components resulting in an increase of the average lifetime. A similar trend with the power of the exciting laser has been observed. These observations are consistent with the dependence of the sensitization magnitude with the total capped nanoparticles concentration (see SI), in which the total excitation (absorbed) power is also varying.

These results are interpreted as a confirmation of the model explained above. The change of the lifetime with the pulse length or laser power represents a direct observation of the energy migration towards the interior of the nanoparticle after sensitization. Indeed, longer excitation pulses, although initially causing an increased population of surface-based excitation, allow for enhanced diffusion towards the inside of the nanoparticle. What is more, it can be employed as a simple way to modulate the ^2^F_5/2_ → ^2^F_7/2_ lifetime, which can be of interest in applications.

Figure 5b shows the I(t) decay curve for the 1030 nm emission, at 460 nm OPO excitation, for the 0.37 mg/ml solution of the chromophore-capped nanoparticle, chromophore/nanoparticle suspension concentration ratio of 1.04. The measurements at the high 2-hydroxyperfluoroanthraquinone/NaYF_4_: Yb^3+^ ratio shows a very different scenario than in the low one. Interestingly, in this case, the average lifetime chromophore-mediated excitation (τ_av_ = 145 μs) is considerably higher than in the lower ligand/NaYF_4_: Yb^3+^ suspension concentration ratio regime and the curve is similar to direct excitation. This is explained as a similar effect to the increase of the total excitation as explained above, as a consequence of the enhanced sensitization, together with a more favorable fluorinated environment for the nanoparticle and screening of the Yb^3+^ from the hydrogenated environment owing to the denser coating. Importantly, these changes occur even for the very short pulses (7 ns). Apart from the advantages derived from the considerable increase of the PL intensity, this result represents another interesting pathway to control the PL lifetime.

## Conclusion

We report an enhancement of the Yb^3+^ NIR emission at 980 nm (1.26 eV) through broad visible light excitation, ranging to wavelengths up to 600 nm in sub 10 nm 2-hydroxy-perfluoroanthraquinone-sensitized Yb^3+^-doped NaYF_4_ nanoparticles in solution. Our system also emits in the 520–750 nm range. We have observed the dependence of the sensitization efficiency with the chromophore/nanoparticle suspension concentration ratio, excitation power and pulse length for the 10% Yb^3+^-doped sample. Our system (for a dense coating of approximately 5.5 ligands per Yb^3+^ ion, or 900 ligands per nanoparticle) provides up to 270 times increase of the overall IR luminescence, as obtained through indirect excitation, with respect to direct Yb^3+^ excitation. This is the highest reported figure to date in an organic-inorganic hybrid NIR emitting lanthanide system, and we assign it to the dense coating with the perfluorinated chromophore and favorable matching of the chromophore states and the Yb^3+^ ion.

We have observed and studied the surface quenching-derived phenomena and their dependence with the preparation and excitation conditions. We have characterized the energy migration leading to the excitation of the Yb^3+^ ions inside the particle, from the excited ones at the surface and described the main channels for excitation dynamics in a hybrid organic-inorganic nano-system and our findings. Our findings allow for a simple strategy for controlling the intensity and modulation of the Yb^3+^ lifetime over a wide range, by tuning the relative importance of the energy transfer channels though physical and chemical parameters.

Our work can be of interest for dual and time-resolved detection and optical or optoelectronic applications and constitutes a description and basic model system for understanding the fundamental processes of the sensitization and energy transfer, with implications towards Er/Yb and Yb/Tm-doped up-converting nanoparticles.

## Methods

Yb^3+^-doped NaYF_4_ synthesis: Y(CF_3_COO)_3_ and Yb(CF_3_COO)_3_ were prepared according to the literature reported method using Y_2_O_3_, Yb_2_O_3_ and trifluoroacetic acid precursors^40^, which were purchased from Sigma-Aldrich and employed without any further purification. The synthesis of sub 10 nm Yb^3+^-doped α-NaYF_4_ nanoparticles followed a modified literature high temperature method^39^: 1-x mmol Y(CF_3_COO)_3_, x mmol Yb(CF_3_COO)_3_ (two samples were prepared, x = 0.2, 0.1) and 2 mmol Na(CF_3_COO) were added to a round bottom flask containing 4 ml oleic acid (Oleic-Ac), 4 ml oleylamine (Oleyl-Am) and 8 ml octadecene (ODE). Initially, the mixture was heated under vacuum (~10 ^2^ bar) at 130 °C for 45 mins to remove the low melting point components and then flushed with N_2_ for 5 mins. The resultant yellow solution was heated in a salt bath, which was preheated to 280 °C, for 40 mins. The final products were isolated by adding ethanol followed by centrifugation. After being washed with ethanol twice, the nanoparticles were dispersed in chloroform. 1-Octadecene (technical grade, 90%), oleic acid (technical grade, 90%), oleylamine (technical grade, 70%) and Na(CF_3_COO) were purchased from Sigma-Aldrich and used without further purification.

Functionalization of Yb^3+^-doped NaYF_4_: 10 mg of the 2-hydroxyperfluoroanthraquinone chromophore were dissolved in 50 ml chloroform, then a portion of the solution was added to a volume of Yb^3+^-doped NaYF_4_ nanoparticle chloroform suspension, according to the desired chromophore to Yb^3+^-doped NaYF_4_ ratio. The color of the mixed solution immediately changed from yellow to pink/orange. The product preparation was significantly clearer than the ligand solution. Potential impurities or unreacted residues were washed with ethanol, which was observed to be transparent to the eye in all produced preparations which implies that there was little unreacted ligand left in the solution. To macroscopically quantify the capping, we have taken into account the corresponding concentration (mass/volume) ratio. We produced preparations with changing overall concentrations of the chromophore-capped nanoparticles from 3.5 mg/ml to 0.19 mg/ml for fixed 2-hydroxyperfluoroanthraquinone/NaYF_4_: Yb^3+^ nanoparticles suspension concentration ratios, as observed by adjusting the dilution of the capped nanoparticles suspension in the chloroform solvent. We also studied different relative chromophore to nanoparticles suspension concentration ratios (ranging from 0.044 to 1.04), produced by fixing the nanoparticle concentration at 0.182 mg/ml, and adding the corresponding proportional mixing of the precursor solution. Binding of the 2-hydroxyperfluoroanthraquinone-derivative ligand to the nanoparticles is suggested by the absence of O-H vibration (Raman spectroscopy) in the dry residue.

Optical measurements: The lipophilic (chloroform solvent) suspensions of organic-functionalized nanoparticles was placed in a 1 × 1 cm^2^ quartz (Q1) cuvette for spectroscopic measurements. For the lifetime and emission measurements, excitation sources included a Continuum Panther optical parametric oscillator (OPO) laser, which was used to provide ~7 ns pulse in the 410–1200 nm range and an electrically modulated 405 nm continuous wavelength (CW) laser was also used. The NIR emission was dispersed in a Triax 550 spectrometer (gratings: 600 lines per mm) and detected by a Hamamatsu R5509-72 photomultiplier. For the excitation spectra measurements, a xenon lamp together with a Jobin-Yvon Horiba Triax 180 spectrometer equipped with 1200 lines per mm gratings was used to get monochromatic excitation. The intensity of the monochromatic light was calibrated by a Newport 918D-UV-OD3R silicon photo detector to normalize the excitation spectra. The fluorescence was measured with the same detector using a 7265 DSP Perkin Elmer Lock-in amplifier.

Structural characterization: Powder X-ray diffraction (XRD) patterns of the dried nanoparticle powder were recorded using Cu Kα radiation (λ = 1.5418 Å). Transmission electron microscopy (TEM) images were taken on a Tecnai FEI T20 microscope operated at 200 KV. Raman spectra were taken with a Jobin-Ybon-Horiba T64000 spectrometer employing an Ar-Kr laser (λ = 647 nm).

## Electronic supplementary material

Supplementary information
