# Supplementary material for: Sensitization, energy transfer and infra-red emission decay modulation in Yb3+-doped NaYF4 nanoparticles with visible light through a perfluoroanthraquinone chromophore
Source: Sci Rep. 2017 Jul 11;7:5066. doi: 10.1038/s41598-017-05350-9 (PMC5505979; doi:10.1038/s41598-017-05350-9)
Supplement: Supplementary file 1 — Supplementary information [file 41598_2017_5350_MOESM1_ESM.pdf]

Supporting information

Sensitization, energy transfer and infra-red emission decay modulation in Yb^{3+}-doped $NaYF_4$ nanoparticles with visible light through a perfluoroanthraquinone chromophore

Haizhou Lu[1,2], Yu Peng[2,3], Huanqing Ye[2,4], Xianjin Cui[5], Jianxu Hu[2], Hang Gu[2], Andrei N. Khlobystov[6], Mark A. Green[7], Philip J. Blower[8], Peter B. Wyatt[3], William P. Gillin[2,9,*], and Ignacio Hernández[10,*]

[1]State Key Laboratory of ASIC and System, SIST, Fudan University, Shanghai, 200433, China.

[2]Materials Research Institute and School of Physics and Astronomy, Queen Mary University of London, Mile End Road, London, E1 4NS, UK.

[3]Materials Research Institute and School of Biological and Chemical Sciences, Queen Mary University of London, Mile End Road, London, E1 4NS, UK.

[4]Division of Physics and Applied Physics, School of Physical and Mathematical Sciences, Nanyang Technological University, 21 Nanyang Link, Singapore 637371

[5]School of Geography, Earth and Environmental Sciences, College of Life Science, University of Birmingham, Edgbaston, Birmingham, B15 2TT, UK.

[6]Nanoscale & Microscale Research Centre (nmRC), University of Nottingham, University Park, Nottingham, NG7 2RD, U.K.

[7]Department of Physics, King's College London, Strand Campus, London, WC2R 2LS, UK.

[8]Division of Imaging Sciences and Biomedical Engineering, King's College London, 4th Floor Lambeth Wing, St Thomas Hospital, London, SE1 7EH, UK.

[9]College of Physical Science and Technology, Sichuan University, Chengdu, 610064, China.

[10]Dpto. CITIMAC, Universidad de Cantabria, Facultad de Ciencias, Avda. Los Castros, s/n 39005, Santander, Spain.

[*]Correspondence to w.gillin@qmul.ac.uk, ignacio.hernandez@unican.es

I. Synthesis and characterization of the 2-hydroxyperfluoroanthraquinone ligand

II. Synthesis and characterization of Cs[YbL_4] complexes, LH = 2-hydroxyperfluoroanthraquinone.

Fig. S1. ^{19}F and ^{1}H NMR spectra of 2-hydroxyperfluoroanthraquinone.

Fig. S2. XRD patterns of the chromophore capped nanoparticles.

Fig. S3. Visible (ligand-based) PL of the chromophore capped nanoparticles. Ligand photoluminescence in the corresponding Yb^{3+} tetrakis complex and triplet emission of the Cs^{+} complex.

Fig. S4. Vis/IR excitation ratio as a function of the suspension concentration for fixed ligand/nanoparticle ratio.

Fig. S5. NIR emission, visible sensitization of Yb^{3+} in the corresponding Cs[YbL_4] complex.

I. Synthesis and characterization of 2-hydroxyperfloroanthraquinone (1,2,3,4,5,6,7-heptafluoro-8-hydroxyanthracene-9,10-dione):

A mixture of anhydrous $AlCl_3$ (3.32 g, 24.9 mmol) and prebaked NaCl (0.73 g, 12.5 mmol) was heated (110 °C) in an oil bath until molten. A mixture of 3,4,5,6-tetrafluorophthaloyl dichloride (**38**) (1.37 g, 5.0 mmol) and 2,3,4-trifluorophenol (0.74 g, 5.0 mmol) was added. The mixture was kept for 3 h at 110 °C under vigorous stirring and then cooled to 0 °C. 3.5 M HCl (10 mL) was added, and the mixture was stirred at 100 °C for 30 min. The reaction mixture was cooled to RT and then diethyl ether (20 mL) was added. The yellow precipitate was filtered off and recrystallized from toluene. 1,2,3,4,5,6,7-Heptafluoro-8-hydroxyanthracene-9,10-dione (0.55 g, 31%) was obtained as yellow needles which sublimed at 280 °C. Main IR peaks, ν_{max}/cm^{-1}: 2688 (br, OH), 1685 (m, C=O), 1651 (m, C=O), 1611, 1507, 1410, 1386, 1279. ^1H NMR, δ_H (400 MHz, $CDCl_3$): 12.61 (s, OH). ^{19}F NMR, δ_F (376 MHz, acetone-d_6): −137.5 (dt, *J* 19, 13 Hz, F-1 or F-4), –140.2 (dt, *J* 19, 12 Hz, F-4 or F-1), −145.5 (td, *J* 20, 12 Hz, F-3 or F-2), −146.4 (dd, *J* 19, 9 Hz, F-5), −147.0 (t, *J* 19 Hz, F-6), −147.5 (td, *J* 20, 11 Hz, F-2 or F-3), −148.2 (dd, *J* 18, 9 Hz, F-7). High resolution mass spectrum: *m/z* (APCI) calcd. for $C_{14}H_2F_7O_3$ ($[M+H]^+$) 350.9887; found 350.9888. [See references 28 or 33 of the main text].

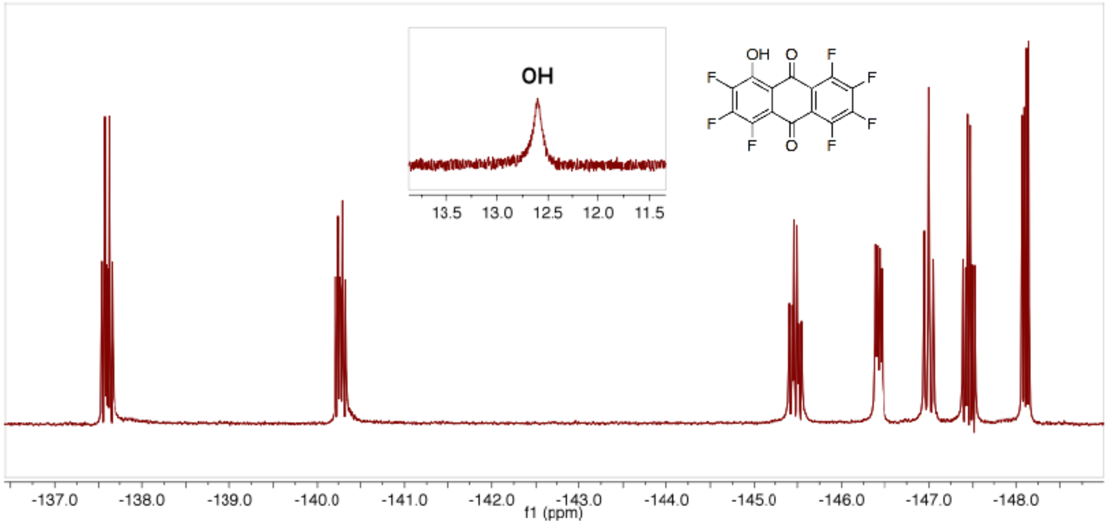

Figure S1. *^{19}F NMR spectrum of 2-hydroxyperfluoroanthraquinone (376 MHz, acetone-d_6) with ^1H NMR spectrum (400 MHz, $CDCl_3$) inset.*

II. Synthesis and characterization of Cs[YbL_4] complexes (LH = 1,2,3,4,5,6,7-heptafluoro-8-hydroxyanthracene-9,10-dione).

An orange/yellow solution of LH = 1,2,3,4,5,6,7-heptafluoro-8-hydroxyanthracene-9,10-dione (290 mg, 0.829 mmol) in CH_2Cl_2 (100 mL) was treated with CsOH (50 wt% in water; 247 mg, 0.824 mmol) in MeOH (2 mL) to give CsL (359 mg, 90%) as a purple precipitate, which was washed with H_2O and CH_2Cl_2.

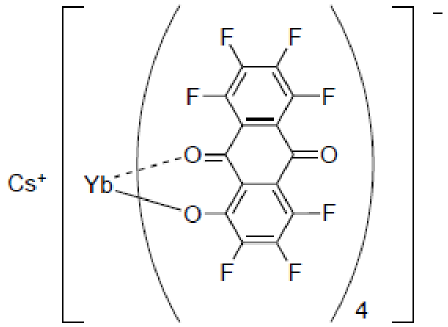

Cs[YbL_4] was synthesized from the CsL salt. CsL (50.0 mg, 0.104 mmol) was dissolved in methanol (50 mL). A solution of $YbCl_3{\cdot}6H_2O$ (10.31 mg, 0.0266 mmol) in methanol (4 mL) was added. The mixture was evaporated to dryness. Acetone was used for extraction of the residue and hexane was added to afford $CsYbL_4$ as an orange-red solid (34 mg, 83%), mp >230 °C (decomp). λ_{max}/nm (MeCN) 466 (ε 21960). Main IR peaks, ν_{max}/cm^{-1}: 1683 (m, C=O), 1641 (s, C=O), 1618, 1599, 1543, 1511, 1495, 1447, 1393. Mass spectrum, *m/z* (MALDI–) calcd. for $C_{56}YbF_{28}O_{12}$ [YbL_4]$^-$: 1569.8; found: 1569.7. Crystallographic structure is found isostructural to that of the analogous Cs[ErL_4] complex (CCDC 905298) [see reference 28 of the main text], the ligand binding at the O,O$^-$ bidentate site.

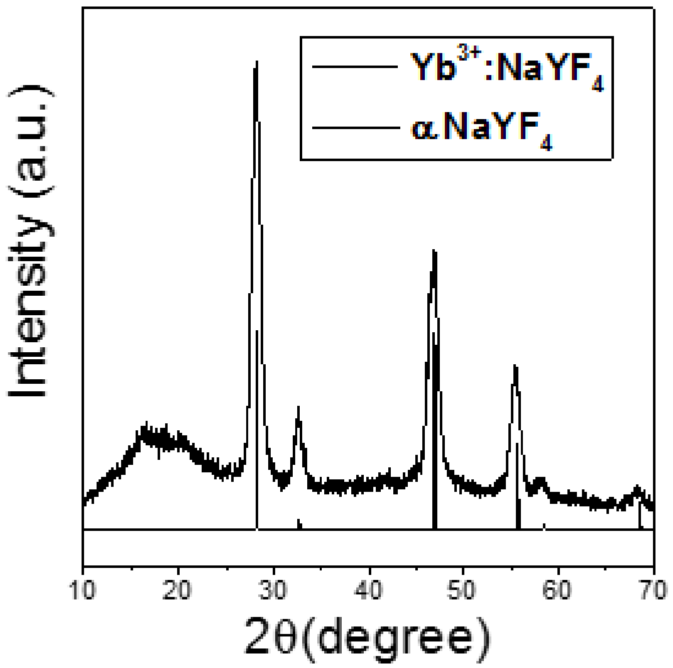

Figure S2. *Measured XRD patterns of α phase 10% Yb^{3+}-doped $NaYF_4$ nanoparticles and calculated line pattern for alpha phase $NaYF_4$ structure. The background (broad feature at $2\theta \sim 20^o$) is assigned to the presence of Oleic-Acid or Oleyl-Amine (solubilizing) ligands, in agreement with reported d-spacing [see, for instance:* Tandon, P., Förster, G. Neubert, R. and Wartewig, S., Phase transitions in oleic acid as studied by X-ray diffraction and FT-Raman spectroscopy, *J. Mol. Structure*, **524,** 201-215 (2000)*]*

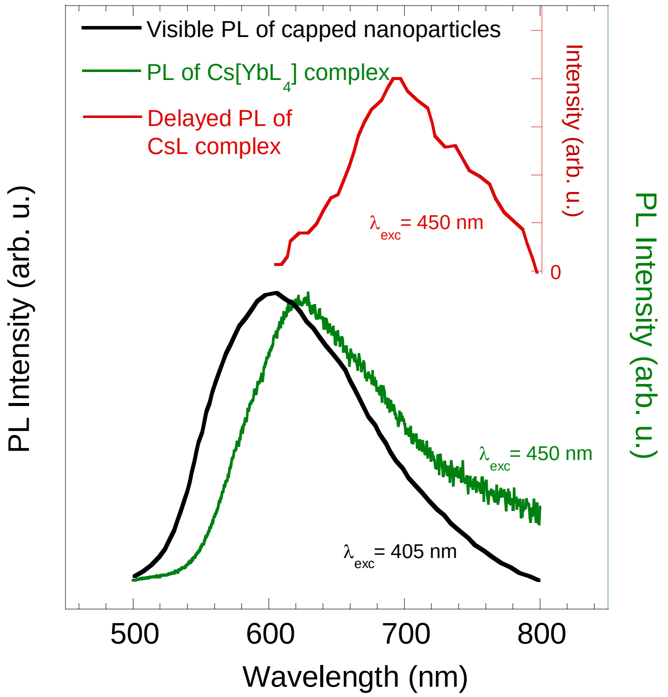

Figure S3. *2-hydroxyperfluoranthraquinone-capped 10% Yb^{3+}-doped $NaYF_4$ nanoparticle emission at 405 nm excitation (black, c/w excitation) represented in comparison with that of the $CsYbL_4$ complex, LH = 2-hydroxyperfluoranthraquinone (green) and the phosphorescence (triplet emission) of the CsL complex (red curve, delay time for PL measurements of 25 μs), obtained for a 5 ns excitation pulse at 450 nm. The spectra are not represented in the same scale. See Figure 3 of the main text for a corresponding level scheme.*

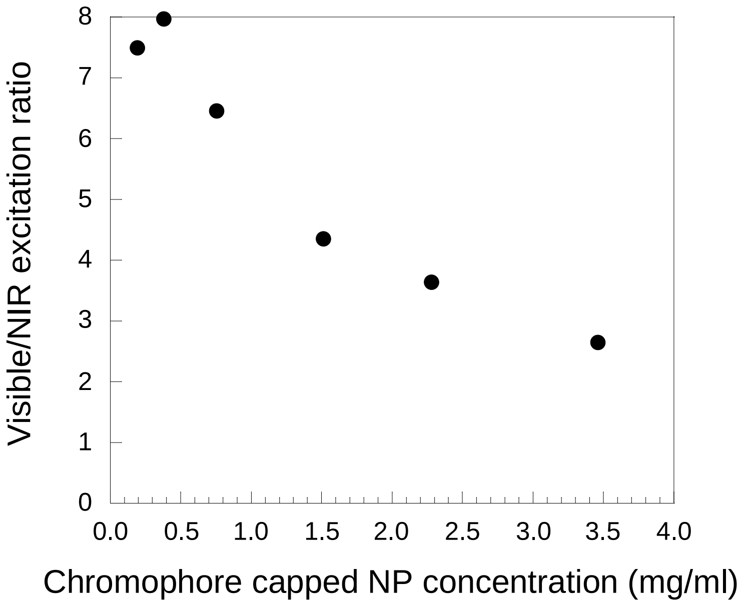

Figure S4. *Excitation ratio, derived from the total PL intensity at 1030 nm for visible (400-700 nm) excitation divided by the intensity for IR excitation (890-1020 nm), as a function of the total capped nanoparticles preparation for a chomophore/NaYF*$_4$*: Yb*$^{3+}$ *nanoparticle suspension concentrations ratio of 0.044.*

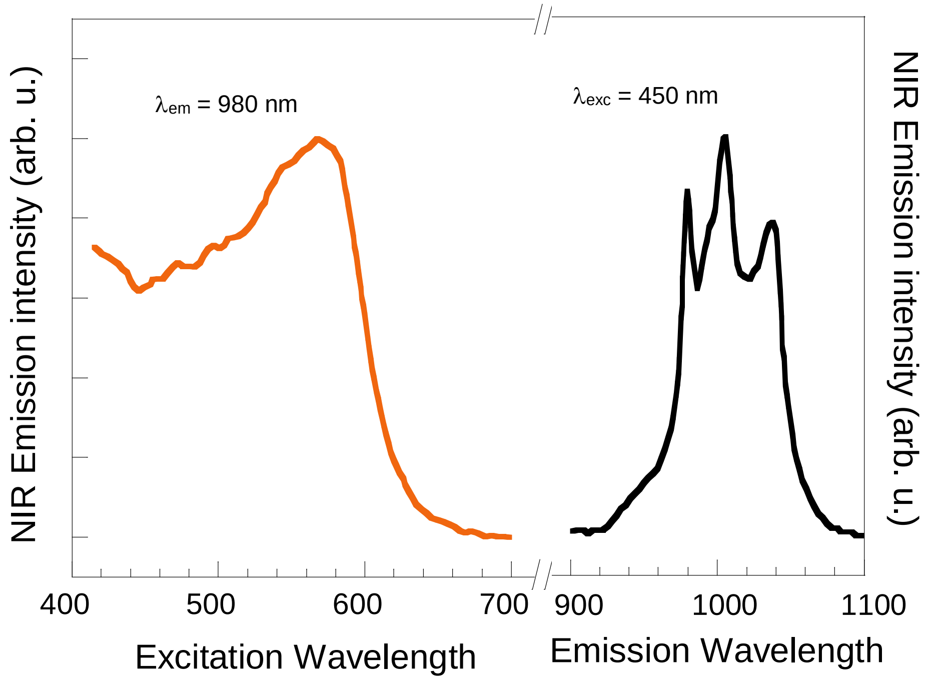

Figure S5. *NIR emission (black curve) and corresponding excitation spectrum (orange) of Cs[YbL*$_4$*] (solid). Note the excitation maximum is redshifted (*$\Delta\lambda_{max} \sim 70$ *nm) with respect to corresponding NIR excitation maximum of the ligand-capped Yb*$^{3+}$*-doped nanoparticles (Fig.3 in the main text). We assign this redshift to the significant stacking interactions within the crystal [see reference 28 of the main text,* Peng, Y. et al., Visible-range sensitization of Er^{3+}-based infrared emission from perfluorinated 2-acylphenoxide complexes, *J. Phys. Chem. Lett.*, **5,** 1560 (2014)*] which depletes the chromophore-based states' energy.*
